# Knowledge, attitude, and preparedness toward IPV care provision among nurses and midwives in Tanzania

**DOI:** 10.1186/s12960-020-00499-3

**Published:** 2020-08-03

**Authors:** Joel Seme Ambikile, Sebalda Leshabari, Mayumi Ohnishi

**Affiliations:** 1 Department of Health Sciences, Nagasaki University Graduate School of Biomedical Sciences, 1-7-1 Sakamoto, Nagasaki-shi, Nagasaki-ken, 8528520 Japan; 2 School of Nursing, Muhimbili University of Health and Allied Sciences, Dar es Salaam, Tanzania

**Keywords:** Intimate partner violence, Care provision, Health professionals

## Abstract

**Background:**

With increasing recognition of intimate partner violence (IPV) as a public health challenge, nurses and midwives are recognized for their crucial role in providing front-line healthcare services for IPV. This study aimed to evaluate knowledge, attitude, and preparedness related to IPV care provision in health facilities among nurses and midwives in Tanzania.

**Methods:**

A self-administered anonymous questionnaire survey was conducted among nurses and midwives working in health facilities in the Mbeya region between December 2018 and January 2019. The questionnaire consisted of questions on their perceived and actual knowledge, attitudes, and preparedness to provide care in relation to IPV.

**Results:**

A total of 662 (50.1%) of 1321 nurses and midwives who worked in hospitals and/or health centers in the Mbeya region participated in this study, and 461 (69.6%) completed questionnaires were included in the analysis. The proportion of nurses and midwives with high scores in IPV perceived knowledge, actual knowledge, attitude, and preparedness to provide care was 59.9%, 53.1%, 54.2%, and 54.0%, respectively. Regardless of the type of facility, gender, educational level, and work experience, the use of IPV guidelines was significantly associated with high scores in IPV perceived knowledge (*P* < 0.001), actual knowledge (*P* = 0.004), and preparedness to provide care (*P* < 0.001), but not attitude, which was negatively associated (*P* = 0.048). Regardless of the type of facility, gender, educational level, and work experience, receiving preservice IPV training was associated with high scores in IPV perceived knowledge (*P* < 0.001), actual knowledge (*P* = 0.049), and preparedness to provide care (*P* = 0.002), but not attitude (*P* = 0.192). Regardless of the type of facility, gender, educational level, and work experience, in-service IPV training was associated with high scores in IPV perceived knowledge (*P* < 0.001), actual knowledge (*P* = 0.043), and preparedness to provide care (*P* = 0.001), but not attitude (*P* = 0.063).

**Conclusions:**

Although guidelines and training could improve nurses’ and midwives’ knowledge and preparedness to provide care regarding IPV, attitudes against IPV care are a challenge. To improve attitudes regarding IPV among front-line nurses and midwives, it is necessary to address concepts of IPV care and sympathy with potential and actual victims of IPV in pre- and in-service training in addition to providing recall-level knowledge.

## Background

Intimate partner violence (IPV) is defined as physical violence, sexual violence, stalking, and psychological aggression (including coercive acts) by a current or former intimate partner [1]. Worldwide, nearly one in three women is affected by IPV, with a higher 12-month prevalence in low-income than high-income countries, where the prevalence is less than 4% [2]. With increased recognition of IPV as a public health problem, the World Health Organization (WHO) has presented effective prevention and intervention strategies for IPV [3]. This requires efforts that target specific risk and protective factors across individual, interpersonal, institutional, community, and societal levels. Such strategies include scaling up IPV services in statewide public health settings, including a patient-centered comprehensive response in clinical settings [4]. In this regard, the role of the health sector in identifying and supporting clients experiencing IPV cannot be overemphasized. This is because IPV clients appear in the healthcare system, but they are not screened and cared for by healthcare providers due to a lack of appropriate capacity; they are also not recognized as victims by healthcare providers [5].

Tanzania has reported a high 12-month IPV prevalence with a national rate of 44% [6]. IPV has serious maternal and neonatal consequences, with direct and indirect pathways that may lead to chronic health problems [7]. Recognition of the importance of addressing gender-based violence (GBV), including IPV, began in 2011 in collaboration with the US President’s Emergency Plan for AIDS Relief. In the same year, the government developed the National Policy Guideline for the Health Sector Prevention and Response to Gender-Based Violence [8], which was instrumental to the development of the National Management Guidelines for the Health Sector Prevention and Response to GBV [9]. Although efforts are being made to integrate GBV care and services into existing service structures, the desired outcomes have not been sufficiently realized to satisfy IPV care and service needs [10]. Some of the reasons attributed to this situation are institutional barriers such as a lack of IPV knowledge, negative attitudes toward IPV, feeling unprepared, a lack of protocols and policies on IPV care provision, and time constraints at health facilities [11–13].

Because IPV is complex and multifaceted, a multisectoral approach is appropriate to address it [14]. One such approach is making IPV care and services available in health care facilities where IPV clients appear but are not recognized by healthcare providers [5]. Nurses and midwives constitute the largest health workforce in Tanzania [15] and are a core aspect of strengthening essential health services at the front line [16]. This puts them in a crucial position to address IPV from a facility-based perspective. IPV knowledge, attitude, and preparedness are important factors that affect nurses’ and midwives’ capacity to manage clients experiencing IPV [17, 18] and are acquired through appropriate preservice and continuing education [19]. Therefore, evaluating nurses’ and midwives’ current conditions related to IPV knowledge, attitudes, and preparedness is important in determining their capacity to provide IPV care and services. This study was performed to evaluate the current conditions related to the knowledge, attitude, and preparedness of nurses and midwives to provide IPV care and services in Tanzania.

## Methods

A cross-sectional study was conducted among nurses and midwives using an anonymous self-administered questionnaire in the Mbeya region, which has 7 districts and a population of three million, between December 2018 and January 2019. The region was selected due to a high lifetime IPV prevalence rate of 67%, which is above the national average of 50.2% [6]. Forty health facilities including 15 hospitals and 25 health centers (HCs) in the Mbeya region were visited for data collection. A total of 1321 nurses and midwives were employed at health facilities at the time of data collection, and 662 (50.1%) nurses who worked at hospitals and/or HCs during the day shift on the data collection day participated in the study. A total of 461 (69.6%) completed questionnaires without missing data on the variables were included in this analysis. Different types of health facility establishments, such as government-, private-, and faith-based establishments, were included in the study. Respondents who were available and who consented at the time/day of data collection were recruited in this study. All shifts were considered for health facilities that had more than one working shift. Nurses and midwives who were not available at the time/day of the data collection due to being off duty, on holiday, or out for official duties and those who could not participate due to various reasons such as being sick and attending to serious patients were excluded from the data collection.

The Physician Readiness to Manage Intimate Partner Violence Survey (PREMIS) tool [20], a widely adopted, adapted, and used tool by various cadres in the health workforce [21–24], was applied to collect data after adjusting for nurses in the study setting. The tool was originally developed in English and translated into Swahili, the common language in Tanzania that most people are more comfortable with. Back translation was performed to ensure the accuracy and quality of the translation by researchers who spoke both languages. The Swahili questionnaire was then pretested among 12 nurses and midwives in two health facilities in different areas of the Mbeya region. Feedback from the pretest was used to clarify the questions, alter the sequence and organization of the questions, and adjust the allotted time for the questionnaire to improve it before data collection. The questionnaire consisted of questions with five parts: demographic characteristics; the Swahili version of PREMIS, including perceived and actual IPV knowledge as the cognitive domain; attitudes toward IPV and preparedness to provide IPV care and services as the affective domain; and the availability and use of guidelines for IPV services. Perceived knowledge of IPV was evaluated by 14 items, and responses were scored from 0 to 2. Actual knowledge of IPV was evaluated by 38 items; appropriate responses were scored as 1 and inappropriate responses as 0. Overall knowledge scores were further calculated by summing the responses; the maximum scores were 28 and 38, respectively. Both knowledge scores were divided into two categories, high (≥ median) and low (< median). Attitude toward IPV was evaluated using 26 items rated on a 5-point Likert scale ranging from 0 (strongly disagree) to 4 (strongly agree). The overall IPV attitude score was calculated by summing the scores of each item; the maximum score was 104. The IPV attitude score was categorized as high (≥ median) or low (< median). Preparedness to provide IPV care and services was assessed using 10 items rated on a 4-point Likert scale ranging from 0 (unprepared) to 3 (quite well prepared). The score for overall preparedness to provide IPV care was calculated by summing the scores of each item; the maximum score was 30. The score for preparedness to provide IPV care and services was categorized as high (≥ median) or low (< median). All categories used were established with reference to a previous related study [25].

IBM SPSS version 20 was used to analyze the data. A chi-square test and logistic regression analysis were used to assess the association between demographic characteristics and IPV service-related knowledge, attitude, and preparedness among nurses. Spearman’s rank correlation coefficient was used to measure the association between perceived IPV knowledge, actual IPV knowledge, IPV attitude, and preparedness to provide IPV services. In all analyses, *P* < 0.05 was used to indicate statistical significance.

## Results

A total of 662 nurses who worked in hospitals and/or HCs during the day shift on the day of data collection participated in the study, which constituted 50.1% of the 1321 nurses in total who were employed in the Mbeya region at the time of data collection. A total of 461 (69.6%) fully completed questionnaires, with no missing data, were included in the analysis.

Table 1 shows the respondents’ demographic information according to the health facility level.

**Table 1:** Demographic characteristics according to the level of health facility (*N* = 461)

	Hospital (*n* = 384)		Health center (*n* = 77)		*P* value
	*n*	%	*n*	%	
Facility ownership					
Government	239	62.2	64	83.1	< 0.001
Non-government	145	37.8	13	16.9	
Gender					
Male	113	29.4	22	28.6	0.880
Female	271	70.6	55	71.4	
Level of education					
Certificate	177	46.1	43	55.8	0.118
Diploma/Bachelor’s/Master’s	207	53.9	34	44.2	
Work experience					
≤ 5 years	116	43.2	29	37.7	0.367
> 5 years	218	56.8	48	62.3	
Use of guidelines^#^					
No	156	40.6	33	42.9	0.716
Yes	228	59.4	44	57.1	
Preservice IPV training					
No/do not remember	266	69.3	63	81.8	0.026
Yes	118	30.7	14	18.2	
In-service IPV training					
No/do not remember	294	76.6	65	84.4	0.130
Yes	90	23.4	12	15.6	

A chi-square test was conducted

^#^Availability and use of standard operating procedure (SOP) or protocol/guideline for IPV services

The respective medians (14, 15, 36, and 14) for IPV perceived knowledge, actual knowledge, attitude, and preparedness to provide IPV care and services were used to categorize respondents with high and low scores. The proportion of respondents who had high IPV perceived knowledge, actual knowledge, attitude, and preparedness to provide IPV care and services was 59.9%, 53.1%, 54.2%, and 54.0%, respectively. The IPV survey tool used was tested for its internal consistency reliability, with a Cronbach’s alpha of 0.758 for all four sets of items. The specific Cronbach’s alphas for IPV perceived knowledge, actual knowledge, attitude, and preparedness to provide IPV care and services were 0.913, 0.691, 0.722, and 0.945, respectively. The proportion of use of IPV guidelines, such as standard operating procedures (SOPs) and protocols, among respondents was 272 (59.0%).

Table 2 demonstrates the Spearman’s rank correlation coefficient between the perceived and actual knowledge, attitude, and preparedness scores.

**Table 2:** Correlations between IPV perceived knowledge, actual knowledge, attitude, and preparedness scores (*N* = 461)

	IPV perceived knowledge		IPV actual knowledge		IPV attitude	
	*ρ*	*P* value	*ρ*	*P* value	*ρ*	*P* value
Perceived IPV knowledge	1					
Actual IPV knowledge	0.171	< 0.001	1			
IPV attitude	− 0.189	< 0.001	− 0.163	< 0.001	1	
Preparedness to provide IPV care	0.408	< 0.001	0.172	< 0.001	− 0.143	0.002

Spearman’s rank correlation coefficient (*ρ*) was calculated

Table 3 shows the associations between demographic characteristics and perceived and actual knowledge, attitude, and preparedness of IPV care provision using the chi-square test.

**Table 3:** Associations between demographic characteristics and IPV perceived knowledge, actual knowledge, attitude, and preparedness scores (*N* = 461)

	IPV perceived knowledge (high ≥ 14)		*P* value	IPV actual knowledge (high ≥ 15)		*P* value	IPV attitude (high ≥ 36)		*P* value	IPV care preparedness (high ≥ 14)		*P* value
	*n*	%		*n*	%		*n*	%		*n*	%	
Level of facility												
Hospital	233	60.7	0.430	193	50.3	0.006	205	53.4	0.416	205	53.4	0.546
Health center	43	55.8		52	67.5		45	58.4		44	57.1	
Facility ownership												
Government	172	56.8	0.060	159	52.5	0.690	170	56.1	0.263	157	51.8	0.190
Non-government	104	65.8		86	54.4		80	50.6		92	58.2	
Gender												
Male	91	67.4	0.034	80	59.3	0.090	71	52.6	0.650	82	60.7	0.062
Female	185	56.7		165	50.6		179	54.9		167	52.1	
Level of education												
Certificate	124	56.4	0.142	116	52.7	0.864	121	55.0	0.751	117	53.2	0.732
Diploma/Bachelor’s/Master’s	152	63.1		129	53.5		129	53.5		132	54.8	
Work experience												
≤ 5 years	127	65.1	0.049	96	49.2	0.149	108	55.4	0.670	108	55.4	0.613
> 5 years	149	56.0		149	56.0		142	53.4		141	53.0	
Use of guidelines^#^												
No	91	48.1	< 0.001	86	45.5	0.006	112	59.3	0.071	82	43.4	< 0.001
Yes	185	68.0		159	58.5		138	50.7		167	61.4	
Preservice IPV training												
No/does not remember	163	59.1	< 0.001	167	68.2	0.105	185	56.2	0.173	162	49.2	0.001
Yes	113	40.9		78	31.8		65	49.2		87	65.9	
In-service IPV training												
No/does not remember	194	54.0	< 0.001	181	50.4	0.028	204	56.8	0.036	179	49.9	0.001
Yes	82	80.4		64	62.7		46	45.1		70	68.6	

A chi-square test was conducted

^#^Availability and use of standard operating procedure (SOP) or protocol/guideline for IPV services

Three models of logistic regression analyses were performed to analyze factors associated with perceived and actual knowledge, attitude, and preparedness for IPV care provision (Table 4). All models included the facility level and facility ownership as the work setting profile and gender, educational level, and work experience as the individual profile for the independent variables in addition to the use of guidelines for model A, preservice training for model B, and in-service training for model C.

**Table 4:** Associations between demographic characteristics and IPV-related perceived and actual knowledge, attitude, and preparedness (*N* = 461)

	IPV perceived knowledge score (high ≥ 14)			IPV actual knowledge score (high ≥ 15)			IPV attitude score (high ≥ 36)			IPV care preparedness score (high ≥ 14)		
	AOR	95% CI	*P*	AOR	95% CI	*P*	AOR	95% CI	*P*	AOR	95% CI	*P*
Model A												
Use of guideline^#^ (ref: no)	2.700	1.802, 4.044	< 0.001	1.786	1.209, 2.639	0.004	0.678	0.462, 0.996	0.048	2.266	1.533, 3.350	< 0.001
Model B												
Preservice IPV training (ref: no/does not remember)	5.793	3.368, 9.967	< 0.001	1.536	1.001, 2.355	0.049	0.757	0.499, 1.150	0.192	1.972	1.279, 3.039	0.002
Model C												
In-service IPV training (ref: no/does not remember)	3.638	2.110, 6.274	< 0.001	1.618	1.016, 2.676	0.043	0.652	0.415, 1.023	0.063	2.209	1.371, 3.560	0.001

Logistic regression analysis was conducted. Level of facility, facility ownership, gender, level of professional education, and work experience were adjusted for in each model

*CI* confidence interval

^#^Availability and use of standard operating procedures (SOPs) or protocols/guidelines for IPV services

## Discussion

The findings of this study demonstrated that in general, nurses had insufficient knowledge, attitudes, and preparedness to provide competent IPV care and services. The level of the IPV cognitive domain, such as knowledge, did not translate into the same level of the affective domain, such as attitude, contrary to previous findings [25]. This study also highlights the role of IPV training and a supportive work environment in the provision of IPV care and services, such as the availability of guidelines regarding IPV care provision, consistent with previous studies conducted within and outside of Tanzania [10, 25–27].

The lack of IPV training experience in this study was consistent with previous studies in various settings [25, 28]. Although insufficient numbers of nurses had the opportunity to receive preservice training on IPV, nurses with shorter work experience, who might be younger and may have recently received nursing education including midwifery education, were more likely to report having preservice training on IPV regardless of educational level. In Tanzania, IPV care provision could be introduced into in-service nursing training even if topics related to IPV are not fully covered during preservice training. Nurses with longer work experience reported being more likely to have had opportunities for in-service training on IPV. Furthermore, nurses with preservice training on IPV were more likely to have had opportunities for in-service training on IPV. There was no association in this study between work experience and the knowledge, attitude, and preparedness scores for IPV care provision. The findings of this study support the role of preservice and in-service IPV training in improving nurses’ capacity to provide IPV services, as corroborated by previous studies [20, 21]. The recently developed WHO IPV curriculum for training healthcare providers [29] needs to be integrated into existing preservice nursing curricula in Tanzania, and the content should include basic/background knowledge on IPV, IPV screening and assessment, offering front-line support to clients who disclose IPV, IPV documentation, IPV referral sources, and orientation to IPV guidelines as per the WHO recommendations [30]. Training on IPV care provision is one of the essential factors to improve nurses’ care capacity; therefore, it is important to integrate IPV care provision into preservice training across the different levels of nursing education and to ensure continuous in-service training, which guarantees and maintains their IPV care capacity and up-to-date knowledge and skills. At the same time, nurses without preservice training on IPV care must become capable of IPV care provision through in-service training because they are front-line health professionals for IPV care. Moreover, this study emphasizes the role of IPV guidelines in supporting the provision of IPV care and services as emphasized by the WHO [31]. In this study, nurses with IPV training experience were more likely to use guidelines. The availability of guidelines and protocols for care provision at health facilities is fundamental, as is the introduction of and training to appropriately utilize guidelines and protocols.

Appropriate and effective IPV educational methods, such as interactive learning and practical applications of learning, may be used [32] after they are adapted to the local context. In this study, there were no associations between educational level, work experience, training opportunities, and the affective domain, such as attitudes toward IPV care. Strengthening the affective domain requires active learning and interactive education, such as case studies, group discussions, role play, and case presentations for affective IPV learning [33], to stimulate the ability to apply, analyze, evaluate, and create interpretations and problem solving.

There are several limitations to this study. First, we did not consider whether the respondents were representative of nurses in Tanzania, although we attempted to recruit respondents from different types of health facilities including hospitals and HCs throughout the region. The demographic characteristics of the study participants from hospitals and HCs likely do not differ from those in other regions in Tanzania. Thus, the findings from this study may demonstrate the actual conditions of IPV-related care capacities in regional areas in Tanzania. Second, only respondents without missing data were included in the final analysis. The excluded respondents may have differed with regard to background information such as educational status, work experience, and IPV training experience. Because of these limitations, the samples we analyzed in this study might be biased toward nurses with higher educational levels. However, there have been limitations in IPV-related care at higher-level health care facilities such as hospitals, which employ nursing personnel with higher educational levels. Thus, conditions of health care provision at dispensaries, which are the lowest level of the health care system in Tanzania and are not addressed in this study, should be extremely limited, including the capacity of nursing personnel and the support system for them. Third, this study was based on self-reported data that may be subjective because we did not observe actual IPV service provision. Therefore, the psychomotor domain was not evaluated in this study, and a comprehensive capacity for IPV care and service provision was not determined because the integration of the cognitive, affective, and psychomotor domains was not analyzed. Future studies should examine the effects of the affective and psychomotor domains on IPV-related care provisions, which would contribute to improving nursing personnel capacity building. Finally, the scores for the internal consistency reliability of the scales used for evaluating actual IPV knowledge and IPV attitude were not sufficiently high. Evaluating actual capacity and performance in providing IPV care requires the use of tools and methodologies to assess the affective and psychomotor domains, including performance tests.

## Conclusions

This study reported actual conditions of knowledge, attitude, and preparedness in relation to IPV care provision among nurses in Tanzania, despite several limitations. The respondents’ attitudes toward IPV care present a challenge for enhancement, although guidelines and training could improve nurses’ knowledge and preparedness to provide care regarding IPV. To strengthen appropriate attitudes toward IPV among front-line nurses, it is necessary to address their thoughts about IPV care and sympathy with potential and actual victims of IPV in pre- and in-service training rather than providing only recall-level knowledge.

## Data Availability

The datasets used and/or analyzed during the current study are available from the corresponding author on reasonable request.

## Abbreviations

AIDS: Acquired immunodeficiency syndrome
AOR: Adjusted odds ratio
CI: Confidence interval
HIV: Human immunodeficiency virus
SOPs: Standard operating procedures
